# Developing a guideline for clinical trial protocol content: Delphi consensus survey

**DOI:** 10.1186/1745-6215-13-176

**Published:** 2012-09-24

**Authors:** Jennifer Marie Tetzlaff, David Moher, An-Wen Chan

**Affiliations:** 1 Ottawa Methods Centre, Clinical Epidemiology Program, Ottawa Hospital Research Institute, 501 Smyth Road, Ottawa, ON K1H 8L6, Canada; 2 Department of Epidemiology and Community Medicine, Faculty of Medicine, University of Ottawa, 451 Smyth Road, Ottawa, ON, K1H 8M5, Canada; 3 Women’s College Research Institute at Women’s College Hospital, University of Toronto, 790 Bay Street, Toronto, ON, M5G 1N8, Canada

**Keywords:** Randomized controlled trials, Delphi consensus survey, SPIRIT Initiative, Protocols, Clinical trials, Reporting guideline

## Abstract

**Background:**

Recent evidence has highlighted deficiencies in clinical trial protocols, having implications for many groups. Existing guidelines for randomized clinical trial (RCT) protocol content vary substantially and most do not describe systematic methodology for their development. As one of three prespecified steps for the systematic development of a guideline for trial protocol content, the objective of this study was to conduct a three-round Delphi consensus survey to develop and refine minimum content for RCT protocols.

**Methods:**

Panellists were identified using a multistep iterative approach, met prespecified minimum criteria and represented key stakeholders who develop or use clinical trial protocols. They were asked to rate concepts for importance in a minimum set of items for RCT protocols. The main outcome measures were degree of importance (scale of 1 to 10; higher scores indicating higher importance) and level of consensus for items. Results were presented as medians, interquartile ranges, counts and percentages.

**Results:**

Ninety-six expert panellists participated in the Delphi consensus survey including trial investigators, methodologists, research ethics board members, funders, industry, regulators and journal editors. Response rates were between 88 and 93% per round. Overall, panellists rated 63 of 88 concepts of high importance (of which 50 had a 25^th^ percentile rating of 8 or greater), 13 of moderate importance (median 6 or 7) and 12 of low importance (median less than or equal to 5) for minimum trial protocol content. General and item-specific comments and subgroup results provided valuable insight for further discussions.

**Conclusions:**

This Delphi process achieved consensus from a large panel of experts from diverse stakeholder groups on essential content for RCT protocols. It also highlights areas of divergence. These results, complemented by other empirical research and consensus meetings, are helping guide the development of a guideline for protocol content.

## Background

The protocol of a randomized clinical trial (RCT) serves many purposes. Protocols provide investigators with a document to guide trial conduct; trial participants with a detailed description of trial methodology; research ethics committees/institutional review boards (REC/IRBs) with a foreknowledge of predefined safeguards to protect participants’ interests and safety; research funders with a means of assessing proposed methods; and systematic reviewers and others with a description of prespecified methods to evaluate potential biases [1-8]. To fulfill these purposes, protocols must be clear, detailed and transparent.

Unfortunately, many protocols do not adequately describe important methodological details such as allocation concealment (59%) [9], primary outcomes (25%) [1], power calculations (27%) [3] and sponsor and investigators’ roles in aspects of trial conduct [10] - all of which have been associated with exaggerated effect sizes and potential bias in trials. The lack of transparency and incomplete description of methods makes critical assessment of trials difficult.

Reporting guidelines have been developed to help improve deficiencies in research reports [11-18]. A recent systematic review examined 40 guidelines for trial protocols; only 20% included any description of their methodological development process. Of those reporting consensus methods, none described formal processes for achieving consensus among stakeholders (for example Nominal consensus technique, Delphi consensus) and none described a systematic consideration of empirical evidence for guideline development [19]. Additionally, recommendations differed considerably across guidelines and many did not include concepts supported by empirical evidence. These inconsistencies and deficiencies have implications for those preparing, using, and reviewing clinical trial protocols.

An international group of researchers launched the SPIRIT (Standard Protocol Items: Recommendations for Interventional Trials) Initiative in 2007, with the primary aim of increasing the transparency and completeness of trial protocols. The main product of this initiative is a checklist of key items to address in protocols of clinical trials. This guideline is being developed with systematic and transparent methodology.

In line with current recommendations [20], three complementary methods were specified *a priori* to develop the SPIRIT checklist: 1) a Delphi consensus survey involving key expert stakeholders in the development and use of clinical trial protocols; 2) a systematic review of empirical evidence supporting the importance of specific checklist items; and 3) face-to-face consensus meetings to develop and finalize the SPIRIT Statement and its associated explanatory document. This paper describes in detail the first component of this research.

## Methods

The objective of this study was to develop and refine minimum content for RCT protocols by expert consensus. We conducted a three-round electronic Delphi survey. Ethics approval was obtained through the Children’s Hospital of Eastern Ontario.

### Selection of participants

Invited expert panellists represented the main stakeholders involved in clinical trials: investigators, methodologists, statisticians and senior study coordinators from academia, pharmaceutical industry and government; REC/IRB members; members of funding and regulatory agencies; and major healthcare journal editors. Experts had to meet the following predefined criteria [21]: relevant knowledge and experience; capacity, willingness and sufficient time to participate; and ability to communicate effectively in English. Participants were selected based on expertise and, where possible, were ranked and selected according to objective criteria (trialists were required to be an author on a minimum of five English-language RCT publications over the past 10 years).

We identified potential panellists using a multistep, iterative approach [22], which included nomination/snowballing, authors of relevant methodological research and the Institute for Scientific Information’s ‘Highly cited researchers in clinical medicine’ [23]. This search was supplemented by specific location-based PubMed searches and targeted Internet searching to increase geographical distribution and areas of panellist expertise. Our objective was to include approximately 100 panellists (40 trialists/clinicians, 20 methodologists, 15 study coordinators, 10 ethics board heads/members, 10 funding/regulatory agency representatives and 5 healthcare journal editors) to enable detection of any divergent opinions between experts groups.

### Selection of preliminary items

An initial list of 59 potential checklist items was collated based on existing protocol guidance [19] and known empirical evidence. Items were grouped under the following broad headings: a) General information; b) Introduction; c) Methods; d) Trial organization and administration; e) Ethical considerations; f) Reporting and dissemination; and g) Other. Each item included a heading and description; wording and structure were kept similar to existing guidelines, where possible.

### Delphi survey

All correspondence occurred via email or facsimile. Approximately two weeks before the survey was administered (August 2007), we informed potential participants of the objectives of the SPIRIT Initiative and Delphi process, and invited them to participate. We solicited reasons for declining, where relevant. Participant anonymity and confidentiality of responses were ensured; individual responses were known only to the moderator (JT). Each survey round was conducted over five to six weeks: one week for pilot testing, three weeks for response acquisition (including two reminders prior to the round closing date) and one week for collating the results and preparing the subsequent round.

Each candidate item was rated in at least two rounds. In each round, respondents were asked to rate items on a 10-point scale (or ‘No judgement’) for their suitability for inclusion in a minimum checklist for RCT protocols. A rating of one corresponded to ‘unimportant - should be dropped as an item to consider’ and ten corresponded to ‘very important - must be included’. We provided panellists with a space following each item and encouraged them to add free text comments, suggest reiterations or suggest additional items they felt would be of benefit for inclusion in the SPIRIT checklist, if relevant. Round 1 also collected demographic information (occupation/field and place of employment) and panellists’ self-rated level of expertise in participating in this process.

**Figure 1:**
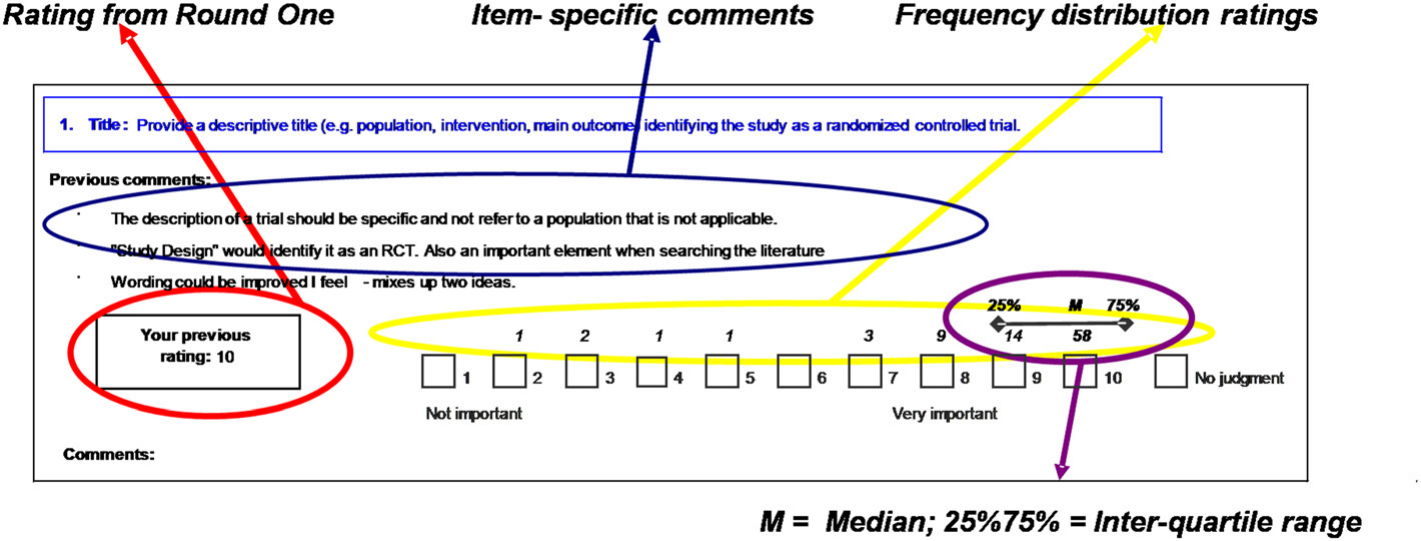
Example of questionnaire layout from Delphi Round 2.

Round 2 of the survey contained all Round 1 items grouped categorically by median scores rounded to the nearest whole number (median ≥ 8; 6 ≤ median ≥ 7; median ≤ 5). No changes were made to checklist items, aside from the addition of newly nominated items from Round 1, which were drafted to include a heading and description; as before, wording and structure were kept similar to existing guidelines, where possible. For each item, panellists were provided with their previous rating, group summary ratings (medians, interquartile ranges (IQRs) and frequency distributions) and anonymized free text comments from Round 1 (Figure 1). They were asked to re-rate the items and respond to existing comments, if desired. Panellists were informed that, following Round 2, consensus would be defined by the consistency of median scores between rounds (median ≥ 8 = high importance, median ≤ 5 = low importance) and the absence of significant issues noted in text comments.

**Figure 2:**
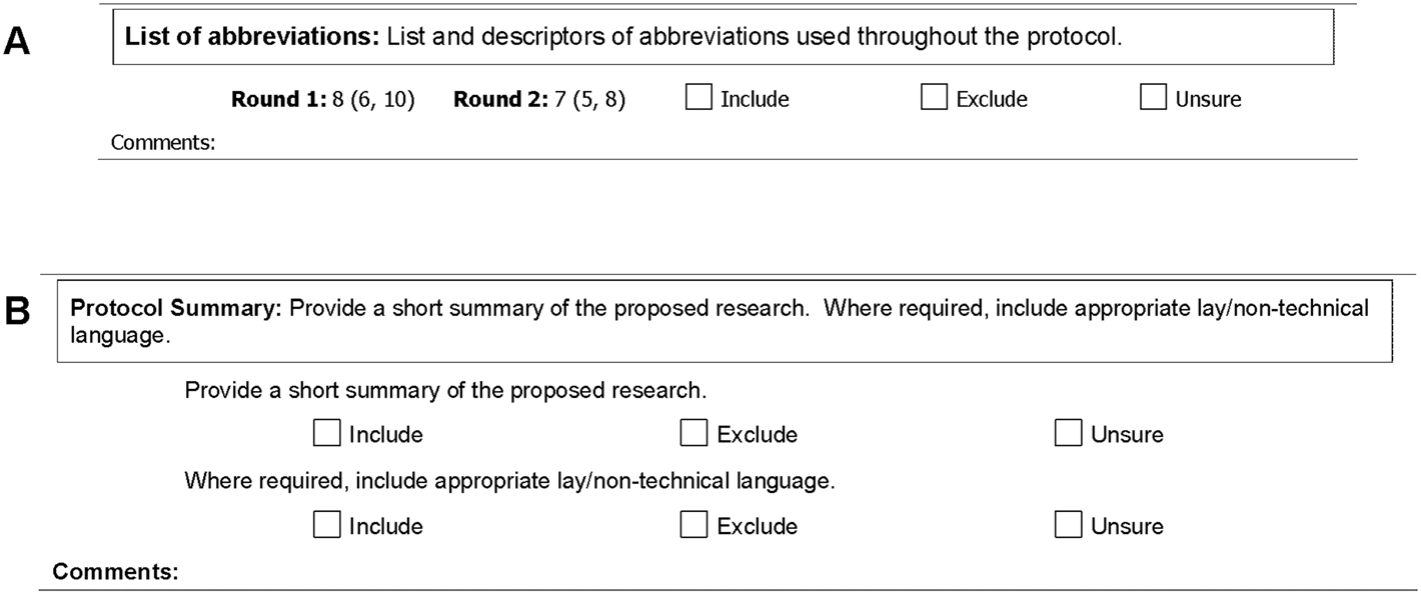
Example of questionnaire layout from Delphi Round 3 Parts 4 (A) and 5 (B).

The third and final round presented results of items reaching consensus (Parts 1 and 2) and three sections requiring additional feedback. Part 3 included items introduced in Round 2 (to be rated as before: from 1 to 10). Parts 4 and 5 included items requiring a third round of feedback: those rated of moderate importance (median 6 to 7) after two rounds (Part 4; Figure 2a) and items where comments suggested that single items contained multiple concepts of differing importance (Part 5; Figure 2b). For the latter, concepts were delineated and respondents were asked to rate each subitem separately. Each item in Parts 4 and 5 had the following response options: ‘Include’, ‘Exclude’ or ‘Unsure’.

### Analysis

Medians and IQRs were calculated for each item. Subgroup analyses were explored by respondents’ occupation and self-rated expertise.

## Results

### Delphi participants

Invitations to participate in the Delphi survey were sent (by email) to 167 experts; we received a response from 123 experts, of which 104 (85%) accepted the invitation. Reasons for declining (n = 19; 15%) were too busy/unable (n = 15), not interested (n = 1) or no reason provided (n = 3). Of the panellists agreeing to participate, eight were unable to respond to either Round 1 or 2 and were not invited to participate in Round 3. Thus, ninety-six experts comprised the final panel.

Panellists met our *a priori* goals for profession/expertise representation (Table 1). Eighty-nine (93%) panellists from 17 countries responded to Round 1; 86 (90%) panellists from 17 countries responded to Round 2; and 84 (88%) panellists from 16 countries responded to Round 3 of the survey. Seventy-seven percent responded to all three rounds, 16% to two rounds and 7% to one round of the Delphi. Most initiated surveys had 100% completion; missing data were sparse and were clarified individually with the respondent.

**Table 1:** Characteristics of Delphi survey panellists (N = 96)

**Question**	**N (%)**
Profession^*^	
Clinical trialist	30 (31)
Healthcare professional	28 (29)
Methodologist	28 (29)
Statistician	16 (17)
Trial coordinator	12 (13)
REC/IRB member	11 (11)
Journal editor	11 (11)
Funding agency representative	5 (5)
Regulatory agency member	3 (3)
Other	7 (7)
Place of employment^*^	
University	58 (60)
Hospital	30 (31)
Government	13 (14)
Non-profit organization	9 (9)
For-profit organization	4 (4)
Self-employed	0 (0)
Other	5 (5)
**Self-perceived level of expertise for survey**	
High level	49 (51)
Mid-high level	33 (34)
Mid level	8 (8)
Low-mid level	1 (1)
Low-level/no expertise	0 (0)

^*^Some panellists selected more than one relevant category. REC/IRB, research ethics committee/institutional review board.

### Delphi results

**Figure 3:**
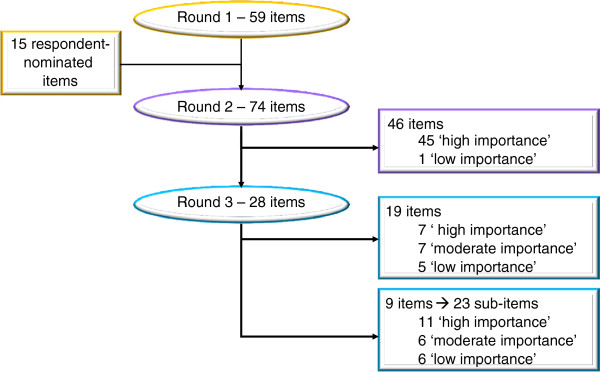
Flow of items through the Delphi survey.

Figure 3 presents the flow of items through the Delphi and Tables 2 and 3 present the final results for each concept. In Round 1, respondents collectively rated 56 of the original 59 items with a median of 8 or greater, three with a median of 6 or 7 (Personnel, Logistics and Budget) and none with a median of 5 or less. All items were recirculated in Round 2, where consensus was achieved for 46 (78%) of the original 59 items; 45 items were considered to be of high importance and 1 (Budget) of low importance. The remaining items - four rated of moderate importance in Round 2 and nine where comments suggested that clarification was necessary - were recirculated for Round 3. Of the four rated of moderate importance, three were ultimately recommended for exclusion (General Approach, Personnel and Logistics). The fifteen panellist-nominated items (rated in Rounds 2 and 3; denoted by § in Tables 2 and 3) included seven with a median of 8 or greater, six with a median of 6 or 7; and two with a median of 5 or less (Signatures, Insurance)

**Table 2:** Consensus - concepts of ‘high importance’ for minimum protocol content following two or three survey rounds

**Section and topic**	Brief description*	**Results**
		**Median (IQR)**
		**or %†**
***General information***		
** Title**	Descriptive title identifying study design	10 (9,10)
** Trial identifier**	Unique number/name and registration information	10 (9,10)
** Protocol version**	Version or amendment number and date	10 (8,10)
** Protocol summary A**‡	Short summary of proposed research	I = 94; E = 5; U = 1
** Names and addresses**	Names/addresses of primary investigators and sponsor	10 (8,10)
** Table of contents**	List of contents and page numbers	8 (5,9)
***Introduction***		
** Rationale**	Outline topic and provide justification for study	10 (9.5, 10)
** Background of the study**	Summary of all previous studies (that is, a SR or reference)	10 (9,10)
** Preliminary data**	Describe preliminary studies (for example by investigators)	9 (8,10)
** Objectives**	Specific objectives and hypotheses for the study	10 (10,10)
** Study location(s) A**‡	Description of intended sites(s)	I = 87; E = 11; U = 2
***Methods***		
*Participants*		
** Population**	Target and study population and source of the latter	10 (9,10)
** Eligibility criteria A**‡	Description of inclusion and exclusion criteria (participants)	I = 99; E = 1; U = 0
** Sample size**	Estimated number; calculations and assumptions	10 (10,10)
** Recruitment**	Process of recruitment (for example advertisements) and enrolment	9 (8,10)
*Design*		
** Type of study**	Description of type/design and trial framework (for example superiority)	10 (10,10)
** Study timeline A**‡	Diagram of participants’ procedures and visits through trial stages	I = 84; E = 10; U = 6
** Sequence generation**	Method used to generate random sequence; details of any restriction	10 (9,10)
** Allocation concealment**	Method used to implement random sequence and whether concealed	10 (10,10)
** Random implementation**	Who will generate sequence, enrol participants and assign to groups	10 (8,10)
** Blinding**	Who (for example participants/investigators/outcome assessors)	10 (10,10)
*Interventions*		
** Interventions A**‡	Precise details; how they will be administered (for example dosage, form)	I = 99; E = 1; U = 0
** Interventions B**‡	Justification of control	I = 87; E = 8; U = 5
** Schedule of interventions**	Number and duration of treatment periods including run-in, washout	10 (10,10)
** Concomitant interventions**	List of relevant treatments permitted or not before or during trial	10 (9,10)
** Risks/Harms**	Known or potential risks for each study intervention	10 (10,10)
*Data collection / management*		
** Outcomes**	Describe and define primary and secondary outcomes	10 (10,10)
** Data collection**	Methods, instruments and timing of data collection and recording	10 (9,10)
** Biological specimens**§	Laboratory evaluation, specimen collection, storage and shipping	8 (6,9)
** Validation of instruments**§	Reliability/validity of instruments or plans to establish validation	8 (6,9)
** Follow-up**	Plans including description and schedule of visits and logistics	10 (9,10)
** Data management**	Plans for data entry, editing, coding and storage	8 (7,9)
** Quality control**	Methods for quality of outcome assessment and data records	9 (8,10)
** Compliance**	Procedures and measures to monitor participant compliance	9 (8,10)
*Statistical methods*		
** Statistical methods**	Methods for primary/secondary outcomes and additional analyses	10 (10,10)
** Withdrawals A**‡	Criteria to withdraw or exclude participants from the intervention	I = 95; E = 2; U = 2
** Withdrawals B**‡	Data to be collected from, and follow-up of, withdrawn participants	I = 85; E = 5; U = 10
** Missing data**	Methods to account for missing or erroneous data	9 (8,10)
** Interim trial monitoring**	Process and timing of any planned interim analyses	10 (9,10)
** Stopping guidelines A**‡	Predefined statistical stopping boundaries	I = 92; E = 6; U = 2
** Stopping guidelines B**‡	Non-statistical criteria for the early trial termination	I = 76;E = 12;U = 12
*Safety and monitoring*		
** Safety evaluations**	Plans for monitoring safety including methods and timing.	10 (9,10)
** DSMB**	If relevant, composition and role of DSMB	9 (9,10)
** Adverse event reporting**	Methods of recording/reporting events; methods to deal with them	10 (9,10)
** Emergency code-breaking**	Establishment/storage of code; when and by whom it can be broken	10 (8,10)
** Trial monitoring**§	Plans and frequency including if independent	8 (6,9)
***Trial organization/administration***		
** Monetary/material support A**‡	Source(s) of financial and material support	I = 94; E = 5; U = 1
** Data ownership**§	Who has ownership; contractual limits for principal investigators	8 (7,10)
***Ethical considerations***		
** Potential benefits and risks**	Potential benefits and risks to participants and society	10 (9,10)
** Agreement and consent**	Method and person responsible; materials for potential participants	10 (9,10)
** Surrogate consent/assent**	Method of obtaining surrogate consent or assent	10 (9,10)
** Confidentiality/Anonymity**	Provisions for protecting personal data and privacy of participants	10 (9,10)
** Ethics approval**	Whether it has been obtained and name of committees	10 (8,10)
** Role of sponsor**	Role of sponsor in design, data collection, analysis, dissemination	10 (8,10)
** Conflict of interest**	Financial or other real or perceived conflicts of interest	10 (8,10)
** Post-trial care**§	Post-trial follow-up, access to treatment, duration; who is responsible	8 (6,9)
***Reporting and dissemination***		
** Protocol amendments**	Methods of communicating to investigators/IRBs and documenting	9 (7,10)
** Dissemination**	How results will be disseminated to participants, practitioners, public	8 (7,10)
** Publication policy**	Who has right to publish; restrictions; authorship guidelines	9 (7,10)
** Reporting of early stopping**§	Dissemination of results if trial is stopped early (for any reason)	8 (5,10)
***Other***		
** Limitations**	Limitations of proposed study, including risk of bias	8 (6,10)
** References**	List of references cited in protocol	10 (9,10)
** Data collection forms**§	Summary table of all forms to be collected at each time point	8 (6,9)

*Abbreviated version of the full description provided to panellists. †Final results from Round 2 or 3; presented as median (IQR) or % Include (I); % Exclude (E); % Unsure (U), as relevant. ‡Subconcepts of original items which required delineation in Round 3. §Concepts added by panellists in Round 1 for rating in Round 2 and 3. DSMB, Data and Safety Monitoring Board; SR, systematic review.

**Table 3:** Concepts of ‘moderate’ or ‘low’ importance for minimum protocol content following two or three survey rounds

**Section and topic**	Brief description*	**Results**
		**Median (IQR)**
		**or %**†
***Rated ‘moderate’ importance***		
**Protocol summary B**‡	Use of lay/non-technical language	I = 63; E = 27; U = 10
**List of abbreviations**‡	List and descriptors of abbreviations used in protocol	I = 74; E = 19; U = 8
**Eligibility criteria B**	Justification of exclusion of subgroups	I = 66; E = 28; U =6
**Monetary/materials support B**‡	List the type(s) of support provided	I = 70; E = 21; U = 10
**Feasibility**§	Acceptability for personnel/participants; capacity for recruitment	6 (3,8)
**Co-enrolment in studies**§	Regulations pertaining to co-enrolment in other research studies	7 (5,8)
**Investigational product(s)**§	Formulation, packaging, labeling and supply; accountability	7 (5,9)
**Pregnancy**§	Monitoring of health of woman and child (short and long term)	7 (4,10)
**Ancillary and substudies**§	Foreseen future uses of data or biological materials; consent	7 (5,9)
**Post-trial data/materials storage**§	Data/materials storage: location(s), duration, responsibility	7 (4,8)
**Appendix materials A**‡	Samples of the standardized case-report forms	I = 65; E = 23; U = 12
**Appendix materials B**‡	Other data collection forms (for example questionnaires)	I = 70; E = 21; U = 10
**Appendix materials C**‡	Consent/assent forms	I = 72; E = 23; U = 5
***Rated ‘low importance’***		
**General approach**‡	Outline the general approach to address the research question	I = 52; E = 42; U = 6
**Study locations B**‡	Briefly justify sites(s) where research is to be conducted	I = 46; E = 46; U = 8
**Study locations C**‡	Relevant demographic/epidemiological information of study region	I = 46; E = 47; U = 8
**Study timeline B**‡	Schematic of the study stages’ expected completion dates	I = 58; E = 30; U = 12
**Withdrawals C**‡	In a multicentre study, when a centre may be discontinued	I = 55; E = 30; U = 16
**Monetary/materials support C**	The amount of support provided	I = 30; E = 57; U = 13
**Monetary/materials support D**‡	How support is provided (for example research account, honorarium)	I = 35; E = 53; U = 12
**Personnel**‡	Names, affiliations, contact details of key trial personnel	I = 40; E = 51; U = 9
**Logistics**‡	Availability of resources incl. administration, equipment, facilities	I = 27; E = 64; U = 10
**Budget**	Budget for personnel, equipment, facilities and supplies	5 (2,6)
**Signatures**§	Signatures including principle investigators or chief medical officer	5 (2,8)
**Insurance**§	Plans including coverage to provide treatment and compensation	5 (2,7)

*Abbreviated version of the full description provided to panellists. †Final results from Round 2 or 3; presented as median (IQR) or % Include (I); % Exclude (E); % Unsure (U), as relevant. ‡Items requiring additional clarification or subconcepts of original items which required delineation in Round 3. §Concepts added by panellists in Round 1 for rating in Round 2 and 3.

Where clarification was required after Round 2 (N = 9 items), panellists’ ratings in Round 3 commonly demonstrated differential support for specific subcomponents (denoted by ‡ in Tables 2 and 3). For example, in general, where items requested specific information plus a justification, respondents strongly favoured the main concept but not the justification (for example *Study locations* (Include (I) = 87%, justification: I = 46%) and *Eligibility criteria* (I = 99%, justification: I = 66%)). The four components of the item *Monetary and material support* also received differing levels of support (source of support: I = 95%; type of support - material, financial: I = 70%; amount of support: I = 30%; how support is provided: I = 35%).

Overall, the Delphi panellists rated 63 concepts of high importance (of which 50 had a lower quartile of 8 or more), 13 of moderate importance and 12 of low importance for inclusion in a minimum set of concepts for RCT protocols (Tables 2 and 3). Most items had narrow IQRs, suggesting agreement between panel members. However, some items had IQRs that spanned from recommendations to exclude the item (five or less) to recommendations to include the item (eight or greater), such as Reporting of early stopping, Ancillary and substudies, trial Feasibility, Signatures and plans to monitor the health of pregnant women and their children (Pregnancy). These items were very often associated with comments stating that the concept is important but is either too specific for recommending in a minimum set for all trials or could be encompassed within another existing item.

#### Summary of text responses

Many general and item-specific comments were received during the three survey rounds and were retained for discussion by the SPIRIT group at subsequent face-to-face consensus meetings; examples are highlighted here.

In general, many respondents stated that, although there were many items, most were important and hence rated highly. While some stated that there must be a ‘balance between guiding researchers and being too prescriptive’, others stated that a comprehensive list is more useful in light of the evidence for poor reporting in protocols and due to the ‘serious business’ of clinical trials that ‘deserve(s) a detailed reporting at any stage’. A few respondents were concerned, however, about the possible increased burden on trialists. Some suggested that some concepts may be addressed in associated documents (for example contracts, statistical and Data and Safety Monitoring Board (DSMB) charters, laboratory manuals) - with reference to such documents in the protocol - or through other sources (for example websites). Finally, some panellists suggested excluding items requiring repeated protocol amendments (for example Personnel, REC/IRB approval) to avoid jeopardizing trial progress with required official amendments and resubmissions.

Other general comments related to ambiguity of the term ‘protocol’, the desired scope of study designs that the checklist should address, and the potential need to define intended users of the protocol or checklist. Some noted that, while all items were potentially important elements, the importance of some may be relative to the target end user.

Item-specific comments consisted mostly of explanations to substantiate chosen ratings, suggested revisions, notes of potential overlap between and opportunities for merging items (for example Background, Rationale and Preliminary data; Risks, Harms and Adverse event reporting), and requests for clarification where items contained more than one concept or were vague. All comments were circulated to panellists in each round and delineations provided, where appropriate, in the final survey round.

#### Subgroup analyses

Subgroup analyses showed few differences between respondents by profession or level of self-perceived expertise (not shown). As examples, REB/IRB members and journals editors were more likely than other groups to support some concepts including a lay summary, a list of abbreviations, and justifications for study locations or eligibility criteria. There were no cases of bimodal results; rather, any differences were in the strength of support with overlapping IQRs. In some cases, the subgroup results enabled examination of the potential validity of additional comments. For example, while some panellists suggested that the items Logistics and Feasibility (which received low support overall) would be important to funding agencies but not to other end users, we found no difference between the opinions of our expert funding agency representatives and other groups for these items. This enabled greater insight and confidence for generating recommendations from the results.

## Discussion

This Delphi survey produced rich information for further development of the SPIRIT Initiative, which aims to develop a guideline for clinical trial protocol content. Recent studies suggest that PubMed indexes over 6,000 RCTs annually [24] and this number has likely increased over time [25]. This finding does not account for trials indexed in other databases (between 20% and 70% of trials depending on the discipline [26]) and the minimum of 40% of trials not reaching full publication [27]. Given that all clinical trials should have a protocol, this Delphi and the SPIRIT Initiative have broad applicability.

Our panellists rated many concepts as highly important for inclusion in RCT protocols, most of which had a strong majority favouring inclusion, indicating consensus within the panel (for example narrow IQRs). The importance of some of these concepts, such as allocation concealment, outcomes (including delineation of primary outcomes), roles of sponsors, and conflicts of interest, are substantiated by strong empirical evidence associating them with risk of bias in trials [2,8,28-34]. Other concepts are supported by more pragmatic, regulatory or ethical rationale. Importantly, many of these concepts are often not described in protocols of RCTs [1,3,9,10,35]. This may be, in part, because most existing protocol content guidelines do not recommend such concepts [19]. The reasons for the variation between existing guidelines and our results are unclear as most guidelines do not report their methods of development.

Our results also indicate where panellists favoured excluding concepts and where a clear consensus was not attained. For the former, such as Budget and Logistics, the lack of support does not suggest that such items should not be included in protocols; only that they may be context-specific (for example not necessary for journal publication of protocols) and thus are not appropriate in a minimum set of requirements. Examples of the latter include items where wide IQRs remained. We believe that a systematic review of the methodological literature is important to complement the Delphi results and to guide and substantiate final recommendations.

Beyond the utility of the Delphi results for trialists, REC/IRBs representatives, funding agencies and the SPIRIT group, this research may be relevant to those developing reporting guidelines and our experience has already helped shape the methodology of other ongoing initiatives. Selecting potential panellists should be given adequate time and attention to ensure they meet the criteria suggested by previous guidance [21] as this is pivotal to both the internal and external validity (generalizability) of the Delphi results. Future endeavours should also consider empirically supported strategies to help increase response rates [36-42] including those used in the current study: survey prenotification/invitation to participate, personalized invitations and surveys, notification of and adherence to expected timelines, clear outline of expectations including time-commitments, written commitment by panellists to participate (reply by email), follow-up reminders to non-respondents, provision of previous rounds’ responses and assurance of confidentiality. We also pilot tested each round, collected panellists’ comments and employed a flexible survey design. Using an Internet-based tool may substantially increase Delphi efficiency and is recommended for future work.

Despite the many benefits of the Delphi consensus technique, the results are only as valid as the opinions of the experts constituting the panel. Even if consensus is attained, validating whether this consensus represents the ‘truth’ is not possible, and we recognize that expert opinion remains among the lowest levels of empirical evidence [43]. To safeguard the validity of our results, we carefully selected a panel representing key stakeholders. Structured, predefined methods were employed to minimize biased response collation. Importantly, our panellists were experienced and committed to completing the process, increasing internal validity of the results.

We chose the Delphi consensus method [21] for this work for several reasons: the research problem was felt to benefit from expert opinion on a collective basis; a larger and more diverse group could be consulted than could effectively meet face-to-face due to expense, size and the logistics of group interaction; and the preservation of participant anonymity allowed for open discussion. This method also shares the advantages of other integrative methods of knowledge translation [44] ideally resulting in a guideline that, beyond being founded on transparent and systematic methods, is externally valid and ultimately meets the needs of end users. We recommend this technique to others embarking on similar initiatives.

## Conclusion

This Delphi consensus has provided a large volume of rich information to guide the development of the SPIRIT checklist, an evidence-based guideline for the content of trial protocols. By applying a formal consensus method and engaging experts from diverse areas, the results of which will be complemented by empirical evidence from the methodological literature, the aim is to collate guidance on important concepts to address in protocols. The SPIRIT Initiative ultimately aspires to help increase transparency and completeness of information in trial protocols, ideally helping to improve the reliability and validity of the medical literature guiding healthcare decisions.

## Abbreviations

CONSORT: Consolidated Standards of Reporting Trials; IQR: interquartile range; RCT: randomized controlled trial; REC/IRB: research ethics committee/institutional review board; SPIRIT: Standard Protocol Items: Recommendations for Interventional Trials.

## Competing interests

The authors have declared that no competing interests exist. All authors are members of the SPIRIT Initiative Steering Group.

## Authors’ contributions

JMT, AWC and DM conceived the study and prepared the initial list of survey items. JMT designed and administered the survey, and collected and analyzed data. JMT wrote the first draft of the manuscript with input from all authors. All authors read and approved the final manuscript.

## Financial disclosure

No direct funding was received for this study. The authors were personally salaried by their institutions during the period of writing (though no specific salary was set aside or given for the writing of this paper). Dr. Moher is supported, in part, by a University (of Ottawa) Research Chair. No funding bodies had any role in the study design, data collection, analysis, decision to publish or preparation of the manuscript.
